# The mirtron miR-1010 functions in concert with its host gene SKIP to balance elevation of nAcRβ2

**DOI:** 10.1038/s41598-020-58655-7

**Published:** 2020-02-03

**Authors:** Christopher Amourda, Timothy E. Saunders

**Affiliations:** 1 Mechanobiology Institute, National University of Singapore, Singapore, Singapore; 2 Department of Biological Sciences, National University of Singapore, Singapore, Singapore; 3 Institute of Molecular and Cell Biology, A*Star, Proteos, Singapore; 4 Present Address: MRC London Institute of Medical Science, Imperial College London, Hammersmith Campus, Du Cane Road, London, W12 0NN UK

**Keywords:** Development, Gene regulation

## Abstract

Mirtrons are non-canonical miRNAs arising by splicing and debranching from short introns. A plethora of introns have been inferred by computational analyses as potential mirtrons. Yet, few have been experimentally validated and their functions, particularly in relation to their host genes, remain poorly understood. Here, we found that *Drosophila* larvae lacking either the mirtron miR-1010 or its binding site in the nicotinic acetylcholine receptor β2 (nAcRβ2) 3′UTR fail to grow properly and pupariate. Increase of cortical nAcRβ2 mediated by neural activity elevates the level of intracellular Ca^2+^, which in turn activates CaMKII and, further downstream, the transcription factor Adf-1. We show that miR-1010 downregulates nAcRβ2. We reveal that Adf-1 initiates the expression of SKIP, the host gene of miR-1010. Preventing synaptic potentials from overshooting their optimal range requires both SKIP to temper synaptic potentials (incoherent feedforward loop) and miR-1010 to reduce nAcRβ2 mRNA levels (negative feedback loop). Our results demonstrate how a mirtron, in coordination with its host gene, contributes to maintaining appropriate receptor levels, which in turn may play a role in maintaining homeostasis.

## Introduction

Since their discovery more than two decades ago^1,2^, microRNAs (miRNAs) have been established as key cellular micromanagers. MiRNAs control nearly all cellular pathways, with a large subset being indispensable to development^3–6^. Canonical miRNAs are genetically encoded short hairpin structures that are recognised, after transcription, by the RNAse type III endonuclease Drosha which cleaves the stem region in the nucleus^7–9^. MiRNAs are subsequently exported from the nucleus and undergo a final step of maturation before being functionally able to regulate the mRNA level of their target genes. Mature miRNAs are 18–22 nucleotides long^3,10–12^. Recent computational and experimental efforts identified a non-canonical miRNA maturation pathway in which introns are debranched from precursor mRNAs by the Lariat debranching enzyme and enter the miRNA processing pathway without requiring cleavage by Drosha^13–15^. This new class of miRNAs were named mirtrons and numerous introns have, subsequently, been inferred as potential mirtrons^16–18^. Further studies revealed that mirtrons are widespread across *taxa* and some show a high degree of conservation^19^, implying important regulatory functions.

The repertoire of canonical miRNA genomic sources is characterised by its versatility. Indeed, miRNAs can be found as single or clustered transcriptional units bearing their own regulatory elements. MiRNAs are also found within introns of host genes both in sense or anti-sense orientations, indicating that their expression does not necessarily correlate with that of their host genes^20–23^. An important question arising from the genomic organisation of canonical miRNAs is: what is the reason for the emergence of mirtrons? It has been hypothesised that alternative miRNA processing pathways may be important in stressful conditions. Anaerobic conditions in tumours or exposure to hormones lead to a down-regulation or inhibition of the miRNA processing components^24,25^. For example, Drosha mRNA level is reduced by around 50% in ovarian-cancer specimens. In such circumstances, pathways regulated by miRNAs are perturbed^24^. Although the presence of mirtrons in such stressful conditions has not been documented, an attractive possibility is that mirtrons fulfil fundamental roles under stressful conditions to maintain cellular homeostasis^19^. In particular, since mirtrons mature regardless of the level of Drosha, their processing and maturation should not be affected under stress as the transcription machinery remains functional. Alternatively, mirtrons may have emerged from mutation of short intronic sequences that evolved into hairpin structures^19,26^. Importantly, the biological significance of intronic miRNAs and, especially, mirtrons must be understood with respect to the function of their host gene^27^.

Here, we show that the mirtron miR-1010 regulates the level of the nicotinic acetylcholine receptor β2 (nAcRβ2) via a negative feedback loop, whereby elevated nAcRβ2 results in increased miR-1010 levels. In the absence of miR-1010 or its binding site within the nAcRβ2 3′UTR, larvae cease growth and do not pupariate. Elevated levels of nAcRβ2 upon neural activity also triggers a homeostatic response whereby SKIP, the host gene of miR-1010, amplifies the Shal K^+^ channel role in tempering membrane potentials^28,29^. However, this negative feedforward response is dispensable, with viable adults emerging in SKIP mutants. Our work demonstrates that miR-1010, transcribed alongside SKIP, is involved in a critical negative feedback loop that regulates nAcRβ2 mRNA levels to maintain balanced expression. Finally, we show that miR-1010 is upregulated upon exposure to nicotine. Therefore, our results could be of interest to studies of nicotine-related disorders.

## Results and Discussion

### MiR-1010, in contrast to its host gene SKIP, is indispensable to viability

**Figure 1:**
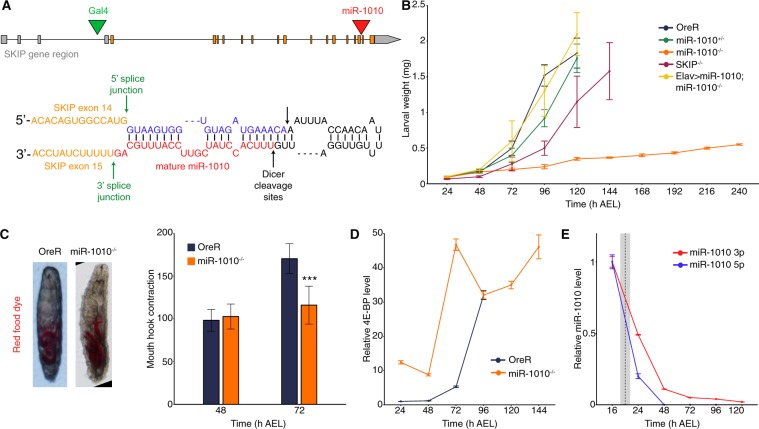
Larvae fail to grow and pupariate in miR-1010^−/−^. (**A**) The mirtron miR-1010 is located in the SKIP gene, between the exons 14 and 15 (red arrowhead, top panel). Green arrowhead shows the insertion point of the Gal4 transposon in the line BL# 62679. MiR-1010 forms a hairpin loop and is debranched from SKIP by the splicing machinery and further matures as a normal miRNA (bottom panel). (**B**) Larval weight was measured directly after hatching until pupariation. OreR, miR-1010^+/−^, SKIP^−/−^ and Elav > miR-1010; miR-1010^−/−^ reached ~2 mg before pupariation. MiR-1010^−/−^ weight was recorded for 10 days but did not pupariate. (**C**) Stereoscope pictures of OreR and miR-1010^−/−^ larvae allowed to eat coloured yeast paste show no feeding behaviour defect (right panel). Mouth hook contraction score in OreR and miR-1010^−/−^. (**D**) Larval 4E-BP transcript levels measured by RT-qPCR in OreR and miR-1010^−/−^. Fold changes are relative to OreR at 24 h AEL. (**E**) Expression level are relative to the expression at 16 h AEL. The dashed line indicates the average hatching time and the grey rectangle represents the standard deviation in hatching times at 25 °C. All values are means ± SD (***P < 0.001, n = at least 9 for each experiment).

MiR-1010 is a mirtron located within the Shal potassium (K^+^) channel Interacting Protein (SKIP) gene, between exons 14 and 15 (Fig. 1A and Supplementary Fig. S1). We found that homozygous mutants for miR-1010 fail to molt and are lethal during the first larval instar. Replacing miR-1010 by a LoxP sequence appears to not affect embryogenesis as larvae hatch without any apparent defects and with similar viability as wild-type animals. We selected late embryos (~16 h) based on the absence of GFP expression from a TM6b, Tb, GFP balancer chromosome. Hence, we cannot discount possible early embryonic defects in miR-1010^−/−^ embryos. From our selected embryos, we note that they hatched with similar survivability to control embryos (~90%). Mutant larvae are unable to grow; they retain a first instar larva size (<0.5 mg) and fail to pupariate even after ten days of larval life (Fig. 1B). Nevertheless, we observed no defects in food intake (as shown by ingestion of red dyed food) or mouth hook contractions in miR-1010^−/−^ larvae (Fig. 1C). We calculated the number of mouth hook contractions per 30 sec^30^. and found no difference between OreR and miR-1010^−/−^ larvae at 48 h AEL. While OreR larvae display an increase of mouth hook contraction at 72 h AEL, miR-1010^−/−^ larvae retain a number of contractions comparable to that of OreR larvae at 48 h AEL. Strikingly, the metabolic brake 4E-BP^31^ shows a 10-fold increase directly at the onset of larval life and remains at a high level in miR-1010^−/−^ larvae as compared to wild-type larvae (Fig. 1D). The high level of 4E-BP is indicative of the larvae experiencing a stressful condition and, as a result, growth is inhibited until returning to more adequate conditions^31^.

Prior to investigating the causes underlying the miR-1010^−/−^ phenotype, we sought to verify that the impeded growth is solely due to the lack of miR-1010 and not a result of an alteration of SKIP. For this purpose, we used a SKIP MiMIC (SKIP^−/−^) mutant where a triple stop codon is inserted within the first intron^32^. Importantly, the MiMIC insertion does not prevent transcription but alters the translation of a given gene. Hence, the miR-1010 level remains at a wild-type level in SKIP^−/−^ larvae (Supplementary Fig. S2A). Concordantly, SKIP^−/−^ larvae show a mild timing phenotype and pupate with a 24 h delay (Fig. 1B). To further validate that miR-1010 is responsible for the larval lethality observed above, we sought to rescue miR-1010^−/−^ larvae by overexpressing UAS-miR-1010 under the control of Elav > Gal4 in a miR-1010 mutant background. We used the driver Elav, a pan-neurally expressed protein, as SKIP is known to be expressed in the nervous system. These larvae managed to develop and eventually pupariate as wild-type larvae (Fig. 1B). These results indicate that the deficit in larval growth is likely due to lack of miR-1010.

### nAcRβ2 is a key target of miR-1010

**Figure 2:**
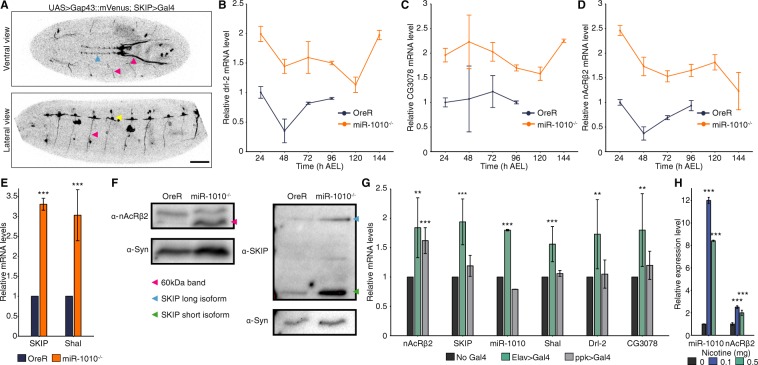
nAcRβ2, Drl-2 and CG3078 levels is elevated in miR-1010^−/−^. (**A**) Confocal imaging of a stage 17 embryo expressing a membrane marker (UAS > Gap43::mVenus) driven by SKIP > Gal4. SKIP/miR-1010 are expressed in the CNS (blue arrowhead), in axons (pink arrowheads) emanating from the CNS and at neuromuscular junctions (yellow arrowhead). Scale bar is 50 µm. (**B**–**D**) Drl-2 (**B**), CG3078 (**C**) and nAcRβ2 (**D**) transcript levels (RT-qPCR) in miR-1010^−/−^ (orange). Fold changes are relative to OreR (dark blue) at 24 h AEL. (**E**) SKIP and Shal transcript levels (RT-qPCR) in miR-1010^−/−^ relative to OreR larvae at 24 h AEL. (**F**) Immunoblot analyses for nAcRβ2 (right panel) and SKIP (left panel) in OreR and miR-1010^−/−^. Syn was used as housekeeping protein. Pink arrowhead indicates the expected size (60 kDa) for nAcRβ2 while the short isoform of SKIP is denoted with a green arrowhead and the full length SKIP protein with a blue arrowhead. nAcRβ2, SKIP and their respective housekeeping protein have different exposure time. Uncropped gel blots are shown in Supplementary Fig. S2F. (**H**) Transcripts levels (RT-qPCR) in nAcRβ2 overexpressed by Elav > Gal4 (green) or ppk > Gal4 (grey) relative to non-induce UAS > nAcRβ2 (black) at 24 h AEL. (**G**) miR-1010 levels upon exposure to 0.1 mg (blue) and 0.5 mg (green) of nicotine as compared to 0 mg (black). All values are means ± SD (*P < 0.05, **P < 0.01, ***P < 0.001, n = at least 9 for each experiment).

We next looked to identify the targets of miR-1010. First, we observed that miR-1010 has its highest expression during late-embryogenesis (16 h AEL) and expression gradually decreases during larval development (Fig. 1E). We used a Gal4 driver inserted downstream of the SKIP regulatory region (SKIP > Gal4, Fig. 1A) to identify potential spatial domains of SKIP and miR-1010 expression. We assume here that SKIP and miR-1010 have the same expression pattern as miR-1010 arises from the debranching of SKIP introns. SKIP and miR-1010 appear to be expressed in the central nervous system (CNS) (Fig. 2A and Supplementary Movie S1), though we note that the SKIP > Gal4 may not drive precisely equivalent expression to the endogenous SKIP protein. However, our results below are not dependent on details of specific target cells. We next screened all targets predicted by the TargetScanFly (v6.2). Amongst 36 tested targets, we found that Drl-2, nAcRβ2 and CG3078 are (i) expressed at meaningful levels (judged by Ct < 33 in qPCR experiments, Supplementary Fig. S2B) and (ii) consistently overexpressed (generally > 2-fold for all targets) in miR-1010^−/−^ throughout larval development (Fig. 2B-D and Supplementary Fig. S2C). Of these, only nAcRβ2 shows higher expression during embryogenesis (Supplementary Fig. S2D). It is, further, the only nicotinic acetylcholine receptor subunit that is upregulated in miR-1010^−/−^ (Supplementary Fig. S2E). Alongside the predicted targets, we noticed that the mRNA levels of SKIP and Shal in miR-1010^−/−^ larvae show a ~3-fold increase as compared to control larvae (Fig. 2E). The increase in mRNA level translates into an increase in protein level for both nAcRβ2 and SKIP (Fig. 2F and Supplementary Fig. S2F). Importantly, the nAcRβ2 protein level is dramatically greater at larval stages in miR-1010^−/−^. SKIP has two shorter isoforms besides its full length protein^29^. SKIP3, the shorter isoform of SKIP, has previously been shown to favour the slow inactivation mode of Shal^29^. We observed an upregulation of both the full length and the shorter isoform, SKIP3, at larval stages (Fig. 2F). To corroborate our results, we checked the mRNA levels of nAcRβ2, Shal, SKIP, Drl-2 and CG3078 in the miR-1010^−/−^ mutant rescued by expressing UAS > miR-1010 with a Elav > Gal4 driver. In this rescue line, nAcRβ2, Shal, Drl-2 and CG3078 are brought down upon overexpression of miR-1010 (Supplementary Fig. S3A and B). However, the SKIP mRNA level is consistently higher (~10-fold) in the rescue line (Supplementary Fig. S3C). This expression pattern is surprising and further experiments will be required to understand the reason underlying SKIP upregulation. Altogether, these results suggest that miR-1010, its host gene, and targets are within the same regulatory pathway.

### Overexpression of nAcRβ2 results in miR-1010 upregulation

Can we rescue miR-1010^−/−^ larvae by knocking down specific targets? Mutants for Drl-2 and CG3078 fail to rescue the miR-1010^−/−^ phenotype (Supplementary Fig. S4A). We were unable to perform similar rescue experiment for the nAcRβ2 as the cytogenic locations of miR-1010 and nAcRβ2 are in close proximity (94A4 and 96A5, respectively) and thus prevent chromosomal recombination. MiRNA deficiency essentially results in overexpression of its targets. We reasoned that overexpressing the coding sequence (*i.e*. without the 3′UTR bearing miR-1010 binding sites) of miR-1010 targets should phenocopy miR-1010^−/−^ (Fig. S4B). We did not impede larval growth as in miR-1010^−/−^ (Supplementary Fig. S4C–E), nevertheless, pan-neural (Elav > Gal4) overexpression of nAcRβ2 resulted in an interesting pattern whereby SKIP, miR-1010 and Shal are upregulated along with Drl-2 and CG3078 (Fig. 2G). On the contrary, overexpression of nAcRβ2 in sensory neurons using Pickpocket Gal4 driver^33,34^ (ppk > Gal4) did not increase the level of SKIP, miR-1010 and Shal (Fig. 2G). These results suggest that miR-1010 acts in specific neurons to regulate nAcRβ2. Further, overexpressing Drl-2 or CG3078 with Elav > Gal4 does not alter the levels of nAcRβ2, SKIP and Shal (Supplementary Fig. S4F,G). These experiments indicate that increased nAcRβ2 levels can give rise to upregulation of the SKIP, miR-1010 and Shal genes.

### MiR-1010/SKIP upregulation is specific to nAcRβ2-mediated increase of neural activity

Among the upregulated targets of miR-1010 that we tested, only nAcRβ2 overexpression resulted in elevated miR-1010 levels. We asked whether miR-1010 expression levels correlate with nAcRβ2 level or more generally with neural activity? To answer this question, we used different means to promote or inhibit synaptic potentials. First, we overexpressed the bacterial sodium channel NaChBac under the control of the Elav > Gal4 driver to boost synaptic potentials^35^. Under these circumstances nAcRβ2 is repressed, possibly to compensate for the increase in neural activity (Supplementary Fig. S5A). MiR-1010 remains near wild-type expression levels and could, therefore, participate in reducing the level of nAcRβ2. To test this hypothesis, we overexpressed NaChBac in a miR-1010 mutant background. In the absence of miR-1010, nAcRβ2 is reduced to a smaller extent (Supplementary Fig. S5A). This indicates that regulation of nAcRβ2 level upon increase in neural activity requires miR-1010. At the same time, SKIP, Shal, Drl-2 and CG3078 expression are downregulated both in the presence or absence of miR-1010 (Supplementary Fig. S5A). These results suggest that the coordinated action of SKIP/miR-1010 is required upon specific elevation of nAcRβ2 levels, but they do not respond to neural stimulation independently of nAcRβ2.

To test this model, we exposed wild-type larvae to different doses (0.1 and 0.5 mg/ml) of nicotine^36^, an agonist of nAcRs, for 24 h. In these larvae, the nAcRβ2 level is increased to 2-fold that of control larvae. We saw a striking ~10-fold increase in miR-1010 levels for both dosage conditions (Fig. 2H). Interestingly, larvae exposed to 0.5 mg/ml of nicotine showed lower miR-1010 expression than larvae exposed to 0.1 mg/ml of nicotine. Larvae exposed to 0.5 mg/ml of nicotine were sluggish and failed to display any response to physical stimuli (touching with a probe), in contrast to larvae exposed to 0.1 mg/ml of nicotine. This result is consistent with a direct relationship between nAcRs activation and miR-1010 expression. Last, we performed the converse experiment and suppressed electrical activity by overexpression of Kir2.1, an inward rectifier K^+^ channel^37^. We noticed a modest increase of nAcRβ2 (1.24-fold increase) whilst SKIP, Shal, Drl-2 and CG3078 remained near control levels (Supplementary Fig. S5B). These results suggest that miR-1010 plays a hitherto underappreciated role in regulating synaptic potentials upon nAcRβ2 activation.

### Removing the miR-1010 binding site in the nAcRβ2 3′UTR phenocopies miR-1010^−/−^

**Figure 3:**
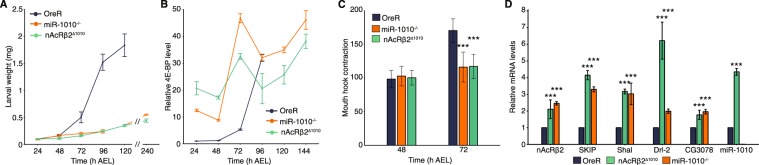
nAcRβ2^∆1010^ phenocopies miR-1010^−/−^. (**A**) Larval weight in nAcRβ2^∆1010^ as compared to OreR and miR-1010^−/−^. (**B**) Larval 4E-BP transcript levels measured by RT-qPCR in OreR, miR-1010^−/−^ and nAcRβ2^∆1010^. Fold changes are relative to OreR at 24 h AEL. (**C**) Mouth hook contraction score in OreR, miR-1010^−/−^, nAcRβ2^∆1010^. (**D**) nAcRβ2, SKIP, Shal, Drl-2, CG3078 and miR-1010 transcript levels (RT-qPCR) in homozygous nAcRβ2^∆1010^ (green) and miR-1010^−/−^ (orange) relative to OreR (dark blue) at 24 h AEL. All values are means ± SD (*P < 0.05, **P < 0.01, ***P < 0.001, n = at least 9 for each experiment).

Our results so far have consistently indicated that the nAcRβ2 is a primary target of miR-1010 and mis-regulation of nAcRβ2 accounts for the phenotype observed in miR-1010 mutant animals. To test this observation more directly, we removed the putative miR-1010 binding site in the 3′UTR of nAcRβ2. We predicted that preventing miR-1010 from regulating the level of the nAcRβ2 should phenocopy miR-1010^−/−^ mutants. The miR-1010 binding site (7-mer) in the nAcRβ2 3′UTR is followed by miR-210.1 and miR-210.2 binding sites. We did not want to interfere with other regulatory elements, therefore, we sought to modify a minimal number of nucleotides in the nAcRβ2 3′UTR binding site. Typically, replacing one or two bases is sufficient to disable the miRNA seed sequence from recognizing its binding site^38,39^. To make this modification, we used the CRISPR-Cas9 method to generate a line, named hereafter nAcRβ2^∆1010^, where the miR-1010 binding site in nAcRβ2 has been modified (Supplementary Figs. S6 and S7). Similar to miR-1010^−/−^, nAcRβ2^∆1010^ complete embryogenesis without apparent defect but they are homozygous lethal in the first larval instar. nAcRβ2^∆1010^ larvae fail to develop and exhibit a growth curve reminiscent to that of miR-1010^−/−^ larvae (<0.5 mg after ten days of larval life, Fig. 3A). This defect in larval growth correlates with the metabolic brake, 4E-BP, being overexpressed at early larval developmental times (Fig. 3B). Therefore, growth is prevented as in miR-1010^−/−^ larvae. Further, mouth-hook contraction numbers are comparable to the pattern observed in miR-1010^−/−^ larvae, with larvae at 72 h AEL retaining features of first instar larvae (Fig. 3C). The resemblance between the miR-1010^−/−^ and nAcRβ2^∆1010^ mutants becomes particularly obvious in light of the expression levels of SKIP, miR-1010, Shal, Drl-2 and CG3078. These molecular players are all upregulated in nAcRβ2^∆1010^, with miR-1010 expression peaking at 5-fold to that of wild-type consistent with nAcRβ2 regulation of miR-1010 expression (Fig. 3D). These results support the hypothesis that miR-1010 regulates nAcRβ2 expression; the loss of miR-1010 regulation of nAcRβ2 phenocopies the lack of growth observed in miR-1010^−/−^.

### Adf-1 controls SKIP, miR-1010 and Shal expression

**Figure 4:**
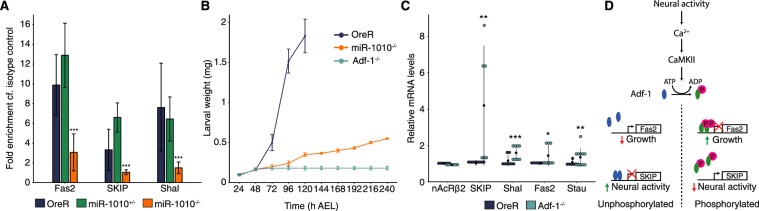
Adf-1 controls the expression of SKIP, miR-1010 and Shal. (**A**) ChIP-qPCR for Adf-1 performed in OreR (dark blue), miR-1010^+/−^ (green) and miR-1010^−/−^ (orange). Fold enrichments are relative to the Rabbit IgG isotype control. (**B**) Larval weight in Adf-1^−/−^ as compared to OreR and miR-1010^−/−^. (**C**) Dot plot representing transcript levels measured in Adf-1^−/−^ (turquoise) relative to OreR (dark blue) at 24 h AEL. Black dots and lines represent, respectively, the mean and the standard deviation for each condition. (**D**) Model of Adf-1-mediated coupling of neural activity and growth. Adf-1 is represented in blue in its non-phosphorylated form and in green upon phosphorylation. All values are means ± SD (*P < 0.05, **P < 0.01, ***P < 0.001, n = at least 9 for each experiments).

We next examined the regulation of SKIP and Shal expression. Through computational analysis, we found that the Alcohol dehydrogenase transcription factor 1 (Adf-1) has predicted binding sequences in both Shal and SKIP regulatory regions^40,41^ (Supplementary Fig. S8A). Further, Adf-1 is phosphorylated by CaMKII, a kinase activated upon neural activity which has been shown to be sufficient to upregulate Shal^28,42^. It has also been demonstrated by ChIP-seq experiments that Adf-1 is phosphorylated upon neural activity and blocks Fas2 and (indirectly) Staufen to allow neuronal growth^42^. We obtained the ChIP-seq results mentioned above and noticed that Adf-1 also binds to Shal and, to a lesser extent, to SKIP (Supplementary Fig. S8B). We confirmed these results by performing a ChIP-qPCR for Adf-1. We used Fas2 as positive control since Adf-1 strongly binds to Fas2 regulatory sequences, as shown by Timmerman and colleagues^42^. Further, Adf-1 binds to Shal and SKIP regulatory regions in OreR and miR-1010^+/−^. However, this binding is significantly reduced in miR-1010^−/−^ (Fig. 4A). Adf-1 has been shown to positively correlate with high Pol II-pausing indices^42^. Therefore, we reasoned that in its non-phosphorylated state Adf-1 is bound to Shal and SKIP regulatory elements and prevents their transcription. Upon neural activity, Adf-1 is phosphorylated and releases Shal and SKIP expression. If our hypothesis is correct, we should then observe higher levels of SKIP, miR-1010 and Shal in Adf-1^−/−^ larvae. The majority of Adf-1^−/−^ (~80%) fail to complete embryogenesis^43^. Those that survive to larval stages do not grow noticeably and display a phenotype reminiscent to that of miR-1010^−/−^ larvae (Fig. 4B). We performed qPCR on Adf-1^−/−^ that reached the larval stage and we saw a general increase in mRNA levels for all candidates, while nAcRβ2 remains at control level (Fig. 4C). However, our results are quite variable, a phenomenon unsurprising given the pleiotropic function of Adf-1^42,43^. Adf-1 promotes neuronal growth upon neural activity by shutting down Fas2 and Staufen. Mechanism(s) must be in place to prevent detrimental overgrowth. By having Adf-1 also regulate Shal and SKIP, the system may couple growth to neural activity. This mechanism introduces an incoherent feedforward loop to ensure robust growth in response to neural activity (Fig. 4D).

## Discussion

nAcRs boost synaptic potentials and trigger the upregulation of cortical nAcRs^28^. These receptors are permeable to Ca^2+^ and the subsequent influx of Ca^2+^ activates CaMKII. In turn, CaMKII triggers the expression of the Shal K^+^ channel. K^+^ ions are subsequently released from neurons and temper membrane potentials. Shal and its interacting protein SKIP have been shown to be important members of a pathway required to stabilise these synaptic potentials^28,29^. Despite the apparent importance of such a pathway, we found that neither knockout of Shal nor SKIP resulted in lethality (Supplementary Fig. S4H). Combining these results, we hypothesised that the mRNA level of nAcRβ2 is downregulated by miR-1010 and that this negative feedback works complementarily to SKIP and Shal to temper synaptic potentials. As previously described, SKIP is presumed to modulate Shal channels inactivation kinetics by favouring Shal slow inactivation mode^29,44,45^. Crucially, however, our results suggest that the SKIP/Shal part of the pathway is only acting to temper the potential response and is not fundamental in restoring the system to a healthy state. In contrast, the negative feedback loop mediated via miR-1010 is indispensable for returning the system to homeostasis after stimulation.

**Figure 5:**
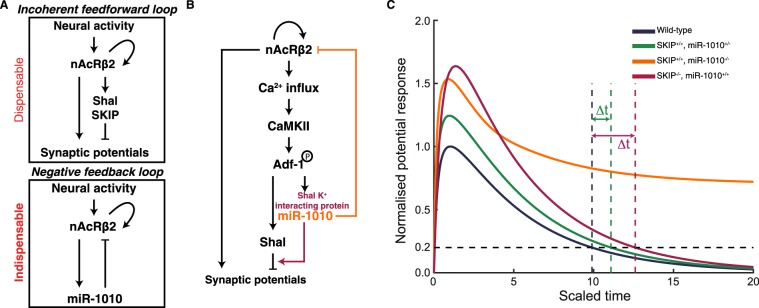
miR-1010 controls synaptic homeostasis by downregulating nAcRβ2 (**A**) nAcRβ2 is part of incoherent feedforward loop to temper synaptic potentials and a negative feedback loop through which miR-1010 downregulates nAcRβ2 upon receptor activation. (**B**) Both loops work cooperatively to prevent synaptic potentials from overshooting their optimal range. (**C**) Long-term average potential response obtained by mass-action kinetic simulation of model outlined in (**A**). Resting potential defined to 0 (corresponding to ~−70meV). Curves normalised to maximum potential in wild-type simulation (parameters and description in Supplementary Note). Time is scaled relative to the lifetime of nAcRβ2. Δt represents delay in potential in miR-1010^+/−^ and SKIP^−/−^ to return to less than 20% of peak response in wild-type conditions.

We have revealed here that nAcRβ2, Shal, SKIP and miR-1010 form a network that contains an incoherent feedforward loop coupled with a negative feedback loop (Fig. 5A). Therefore, we posit that the mirtron miR-1010 and its host gene SKIP are working in tandem to ensure synaptic homeostasis (Fig. 5B). It will be interesting to test this hypothesis using patch-clamp recordings. However, in the first instar larvae such experiments have proven to be challenging. As a simple test of our model, we have used mass-action kinetics to model the average long-term voltage potential response due to the two interaction loops outlined in Fig. 5A after activation of nAcRβ2 (see Supplementary Note for details). Our objective here is to explore whether the regulatory logic defined by the feedforward and feedback loops is consistent with the experimental observations. We see that loss of SKIP or partial loss of miR-1010 results in delayed return of the potential to homeostatic levels (Fig. 5C), qualitatively consistent with our observed delay in developmental time (Fig. 1B). However, complete loss of miR-1010 results in the potential staying active over a long period (Fig. 5C), consistent with the observed stress response seen in miR-1010^−/−^. From our experiments, and supported by the simulations, we conclude that the expression level of miR-1010 is effectively acting like a switch, controlling whether nAcRβ2 has high or low expression (see Supplementary Note for further discussion). Interestingly, the simulation predicts that the average membrane potential response will be higher in miR-1010^+/−^ and SKIP^−/−^ as compared with wild-type larvae. As noted above, patch clamp recordings will be needed to test this prediction, but such experiments are currently very challenging. Our results point to the importance of miR-1010 in maintaining homeostasis upon nAcRβ2-mediated neural activity. Tempering synaptic potentials through an incoherent feedforward loop are not sufficient to restore membrane potentials to an optimal range. The concomitant downregulation of the transmembrane receptor, as exemplified by miR-1010 regulation of nAcRβ2, is indispensable to restore equilibrium.

The upregulation of Drl-2 and CG3078 upon overexpression of nAcRβ2 is intriguing. The most likely explanation is that the higher levels of Drl-2 and CG3078 in miR-1010^−/−^ larvae is simply a response to increased neural activity, not the direct result of a lack of miR-1010. CG3078 upregulation upon neural activity is plausible as its inferred function is to regulate K^+^ channel activity^46^. However, the reason for Drl-2, an axon guiding protein^47–49^, upregulation is more enigmatic; it may be a response to axonal growth following increased activity. Moreover, given the pleiotropic effects of CaMKII and Adf-1, deciphering the specificity underlying the miR-1010-mediated regulation of nAcRβ2 constitutes another exciting avenue for future investigations.

We have studied the role of miR-1010 in a developmental context but it is important to note that exposure to nicotine results in a dramatic increase of miR-1010 levels in wild-type larvae. Hence, this pathway may not be restricted to development and could apply to later stages of life. Deciphering the relevance of miR-1010 regulation in nicotine-related disorders (such as nicotine addiction or Alzheimer disease) offers exciting avenues of future research. Our work sheds light on the coherence between the functions of mirtrons and their host genes, in particular that SKIP and miR-1010 act in a coordinated fashion. We predict that (i) such regulatory loops are not restricted to synaptic homeostasis and (ii) homologous pathways exist in higher organisms. MiR-1010 shares sequence homologies with the mammalian miR-412^50^. However, miR-412 is not a mirtron and does not seem to directly regulate nAcRs (from computationally predicted targets). Identifying a functional homolog would help to further understand the pertinence of homeostasis control by mirtrons.

## Materials and Methods

### Fly stocks

OregonR (OreR) was used as wild-type. MiR-1010^−/−^ (w[*]; TI{TI}mir-1010[KO]/TM3, P{w[ + mC] = GAL4-twi.G}2.3, P{UAS-2xEGFP}AH2.3, Sb[1] Ser[1]) was generated in the Cohen lab by homologous recombination and obtained from Bloomington (BL#58886)^51^. MiR-1010^KO^/TM3 was crossed to TM3/TM6b, GFP (modified from BL#4887) to generate miR-1010^KO^/TM6b, GFP. GFP-negative animals are miR-1010^−/−^. Further SKIP^−/−^ (y[1] w[67c23]; Mi{ET1}SKIP[MB00109], BL#22699), Shal^−/−^ (y[1] w[*]; Mi{y[ + mDint2] = MIC}Shal[MI00446], BL#31006), Drl-2^−/−^ (w[1118]; Mi{ET1}Drl-2[MB06584], BL#25627), CG3078^−/−^ (Mi{ET1}CG3078[MB01438] w[1118], BL#23185) and Adf-1^−/−^ (y[1] w[67c23]; P{w[ + mC] = lacW}Adf1[k14805]/CyO, BL#11135) mutant were used and all obtained from Bloomington. Overexpression experiments were carried out with Elav > Gal4 (P{w[ + mC] = GAL4-elav.L}2/CyO, BL#8765) and ppk > Gal4 (w[*]; P{w[ + mC] = ppk-GAL4.G}2, BL#32078) to drive UAS > Drl-2 × 2 (w[*] P{w[ + mC] = UAS-Drl-2.S}1a P{w[ + mC] = UAS-Drl-2.S}1b, BL#64297), UAS > NaChBac (y[1] w[*]; P{w[ + mC] = UAS-NaChBac}2, BL#9469), UAS > Kir2.1 (w[*]; P{w[ + mC] = UAS-Hsap\KCNJ2.EGFP}7, BL#6595), UAS > miR-1010 (w[*]; PBac{y[ + mDint2] w[ + mC] = UAS-mir-1010.S}VK00037/CyO, BL#60663), SKIP > Gal4 (w[1118]; PBac{w[ + mC] = IT.GAL4}SKIP[0165-G4], BL#62679), w[1118]; UAS > nAcRβ2 and w[1118]; UAS > CG3078 (the latter two were made in the lab). The line w[1118]; nAcRβ2^∆1010^ mutant line was made for this study by CRISPR-Cas9 modification. In all experiments adult flies, embryos and larvae were maintained at 25 °C.

### Transgenic line generation

Ten embryos late were harvested and their mRNA was isolated using the mRNA using the RNeasy mini kit (Qiagen). The cDNA was obtained with the SuperScript III Reverse Transcriptase (Thermo Fisher Scientific). Subsequently, PCR primers were designed to amplify the coding sequence of nAcRβ2 and CG3078. The PCR products were inserted into the pENTR/D-TOPO vector (Invitrogen). The PCR products were recombined into the pPW vector (containing the UAS promoter) using the LR clonase (Invitrogen). The resulting plasmids (pPW-nAcRβ2 and pPW-CG3078) were injected into embryos by BestGene Inc. The CRISPR-Cas9 nAcRβ2 3′UTR mutant was generated using the protocol described at flyCRISPR (http://flycrispr.molbio.wisc.edu/). Particularly, a PAM site was identified upstream of the miR-1010 binding site within the nAcRβ2 3′UTR. A 137 bp single stranded oligonucleotide (ssODN) bearing mutations in the miR-1010 binding site and synthetized. The targeting gRNA was constructed in using the pU6-BbsI-chiRNA vector via the BbsI restriction sites. A mixture containing the ssODN and the gRNA was injected into the BDSC#56552 line by BestGene Inc.

### Weighing larvae

Larvae were collected from hatching (i.e 24 h after egg laying) and weighed every 24 h until pupariation. Larvae were reared at ~10 animals per plate in apple juice plates supplemented with standard yeast paste. Different concentrations of nicotine (Sigma) was added to the yeast paste in relevant experiments. Groups of 5 larvae were weighed at the indicated time AEL with an Ohaus Precision balance. Ten independent batches were weighed to buffer the effect from environmental conditions.

### Feeding behaviour assay

Newly hatch OreR or miR-1010^−/−^ larvae were exposed to standard yeast paste supplemented with red food dye (Star Brand). Larvae were allowed to feed for 1 h before being imaged. Larvae were imaged on a Nikon SMZ18 stereomicrosope. Mouth hook contraction was measured as described in^30^. Briefly, synchronised larvae were transferred to a liquid solution containing 1% of yeast extract in water. The number of mouth-hook contractions per 30 seconds was recorded for 20 larvae per genotype.

### Live imaging on a confocal microscope

Embryos (UAS > Gap43::mVenus;SKIP > Gal4) were collected, dechorionated using household bleach and mounted on a MatTek dish. The embryos were imaged on a Zeiss LSM710 microscope with a C-Apochromat 32 × /0.85 NA water objective. The pixel size is 830 nm and the image resolution is 512 × 512 pixels. At each time point a stack of 20 images separated by 3 µm was acquired. The temporal resolution was 6 minutes. Each Z-stack for each time point was Z-projected (maximum intensity) and the colours were inverted.

### qPCR on mRNA and miRNA

Ten non-dechorionated embryos were aligned on an apple juice agar plate and allowed to develop at 25 °C from the blastoderm stage until reaching the desired stage. Embryos were subsequently harvested. mRNA was isolated using the mRNA using the RNeasy mini kit (Qiagen). The cDNA was obtained with the SuperScript III Reverse Transcriptase (Thermo Fisher Scientific). Real-time quantitative PCR was performed using SYBR® Green Assay (Thermo Fisher Scientific) on a Bio-Rad CFX96 Real-time system following the manufacturer’s recommendations. The Rpl32 was used as endogenous control. MiRNAs were isolated using the miRNeasy mini kit (Qiagen). The reverse transcription was perform with miRNA-specific Taqman RT primers (Thermo Fisher Scientific) and reverse transcriptase (Thermo Fisher Scientific). The real-time quantitative PCR was performed with miRNA-specific Taqman MGB probes and Taqman Universal Master Mix II (Thermo Fisher Scientific) on a Bio-Rad CFX96 Real-time system. The U27 was used as endogenous control. Three independent replicates have been performed for each primer sets and the significance was calculated with a standard t-test.

### Immunoblot analyses

Typical 10 larvae aged at 25 °C were grinded in 100 µl of RIPA buffer (Sigma) supplemented Proteinase Cocktail Inhibitor (1/100, Sigma). Protein concentration was measured by performing a Bradford assay: 30 µl of lysate were added to 1.5 ml of Bradford reagent (BioRad). Absorbance at 595 nm was measured and dilutions of BSA were used to make the standard curve. For each blot, equal amount of total protein were loaded for both OreR and miR-1010^−/−^ (between 20 to 40 µg of proteins). The electrophoresis and transfer on PSDF membrane were performed using standard procedures. Subsequently, the membrane was blocked in 5% BSA for 1 h at RT on a rocking platform. The primary antibody was incubated with the membrane O/N at 4 °C on a rocking platform. The membrane was washed 3x in PBS-0.1% Tween before incubation with the secondary antibody for 1 h at RT on a rocking platform. The signal was revealed with ECL and recorded on a BioRad ChemiDoc Imaging System. In a given gel, the lanes were exposed for the same duration and applied with same brightness and contrast settings. We used α-Syn (DSHB 8C3, 1/500) as loading control. Further, we generated SKIP (1/500) and nAcRbβ2 (1/500) with the help of Absea Biotechnology Ltd. The antibodies were generated using, respectively, the peptides CETPPNNELELVLRE (previously published^25^) and CMRRTQYTLPDYDDSTPSNGYTNEIDVR. Three independent replicates have been performed for each blot.

### Chromatin immunoprecipitation and qPCR

Late embryos aged at 25 °C were dechorionated in household bleach. Embryos were crosslinked for 15 minutes in a solution containing 2 ml of PBS, 6 ml of Heptane and 180 µl of 20% paraformaldehyde. Embryos were transferred to a 1.5 ml tube and the crosslinking was quenched with the addition of 125 mM glycine in PBS 15 min after the start of fixation. ChIP samples were essentially prepared as described in Blythe and Wieschaus, 2015^52^. Sonication was performed on a Sartorius stedim Labsonic® M with a microtip horn. An input control corresponding to 2% of the volume per reaction was taken after sonication. Immunoprecipatations (IPs) were performed with an Adf-1 antibody (courtesy of Victor Corces) for 15 hours at 4 °C. ChIPped DNA were extracted with a Qiaquick spin column (Qiagen). Real-time quantitative PCR was performed using SYBR® Green Assay (Thermo Fisher Scientific) on a Bio-Rad CFX96 Real-time system. Primers pairs were designed using the GeneScript primer design tool from sequences obtained from ChIP-seq data. The fold enrichment as compared to the Rabbit IgG isotype control (Thermo Fisher Scientific) was used to represent ChIP-qPCR data. Three independent replicates have been performed for each primer sets and the significance was calculated with a standard t-test.

### Statistical analysis

Data are presented as Mean ± SD for histograms. Data were first subjected to a Bartlett test to assess the homogeneity of variances. In the absence of significance difference, Student’s t-tests were used to calculated the p-value. Otherwise Welch’s t-tests was used for unequal variances. All data were plotted and analysed in R.

### Mathematical model

See Supplementary note for details of modelling and simulations.

### Ethics statement

The work carried in this study is in compliance with the ethical rules in place at the National University of Singapore.

## Supplementary information

Supplementary information.

Supplementary Movie S1. SKIP/miR-1010 expression across embryogenesis.
