# Supplementary material for: The mirtron miR-1010 functions in concert with its host gene SKIP to balance elevation of nAcRβ2
Source: Sci Rep. 2020 Feb 3;10:1688. doi: 10.1038/s41598-020-58655-7 (PMC6997181; doi:10.1038/s41598-020-58655-7)
Supplement: Supplementary file 1 — Supplementary information. [file 41598_2020_58655_MOESM1_ESM.pdf]

The mirtron miR-1010 functions in concert with its host gene SKIP to balance elevation of nAcRβ2

Christopher Amourda[1,4,*,#] and Timothy E. Saunders[1,2,3,*]

Supplementary figures

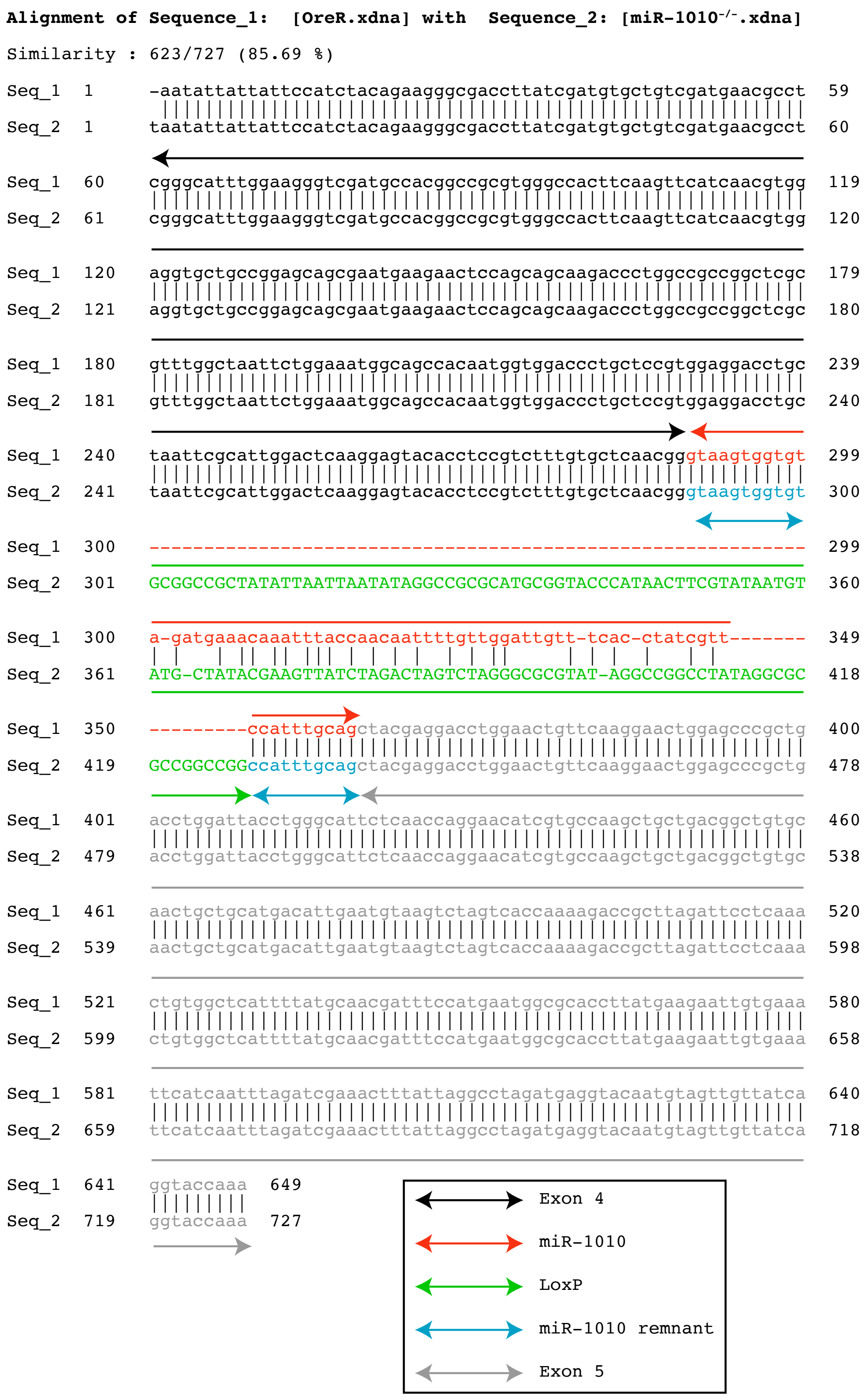

Supplementary Figure S1. Genomic sequencing of the SKIP gene in OreR and miR-1010$^{-/-}$
The miR-1010 locus was sequenced in miR-1010$^{-/-}$ larvae and aligned against OreR sequencing results. The exons 4 (black) and 5 (grey) remains unaffected. MiR-1010 (red) has been almost entirely removed and replaced by a LoxP sequence (green) in miR-1010$^{-/-}$ animals. 5' and 3' remnants of miR-1010 subsist in miR-1010$^{-/-}$ to aid splicing of the SKIP gene.

A

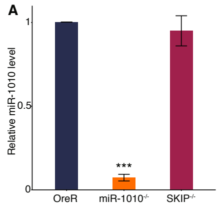

B

Genes	Average Ct value
Axo	26.47
CG10479	29.86
CG12488	28.92
GABA-B-R1	27.71
GalNAc	23.53
Mef2	22.06
Rab3	22.81
Ten-m	24.40
beat-IIa	26.15
bftz-f1	26.08
BG642312	32.39
CG11776	30.75
CG12069	31.89
CG12508	28.94
CG13575	31.28
CG14317	34.73
CG15414	30.17
CG15471	33.77
CG30083	33.61
CG30111	25.16
CG3020	28.84
CG32406	27.01
CG4461	32.40
CG5758	26.10
CG6108	25.87
CG6982	25.80
CG8757	32.67
Gr28b	31.09
Obp56g	34.32
Osi5	32.68
dpr8	30.77
nxf4	31.83
m8	28.37

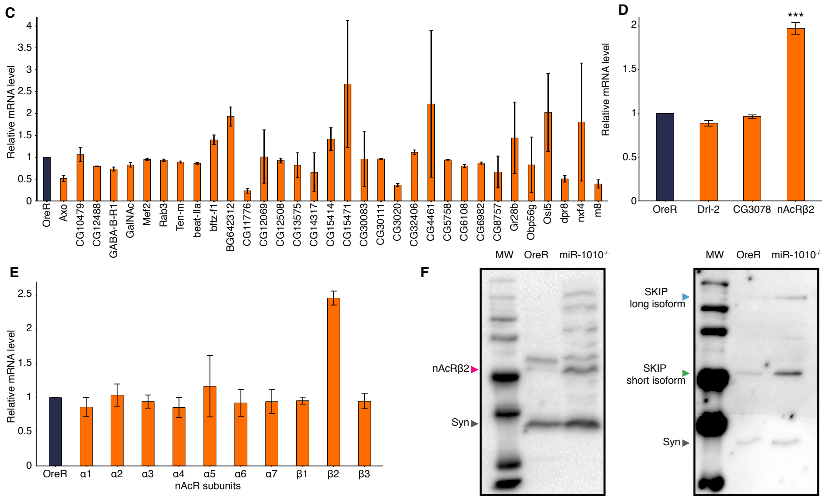

Supplementary Figure S2. Expression level of miR-1010 and its targets in miR-1010$^{-/-}$

(A) MiR-1010 level measured in miR-1010$^{-/-}$ and in SKIP$^{-/-}$ larvae relative to OreR at 24h AEL. All values are means ± SD (*P<0.05, **P<0.01, ***P<0.001, n = at least 9 for each experiments).

(**B**) Average Ct values obtained for miR-1010 targets.

(C) Larval miR-1010 targets transcript levels measured by RT-qPCR in OreR (dark blue) and miR-1010$^{-/-}$ (orange). Fold changes are relative OreR at 24h AEL.

(D) Relative mRNA levels (RT-qPCR) for Drl-2, CG3078 and nAcRβ2 in miR-1010-/- (orange) as compared to OreR (dark blue) during late embryogenesis.

(E) Larval nAcR subtypes expression in miR-1010$^{-/-}$ (orange) relative to OreR (dark blue) at 24h AEL. All values are means ± SD (*P<0.05, **P<0.01, ***P<0.001, n = at least 9 for each experiments).

(**F**) Full-length immunoblots of nAcRβ2 and SKIP in OreR and miR-1010$^{-/-}$ shown in Fig. 2F.

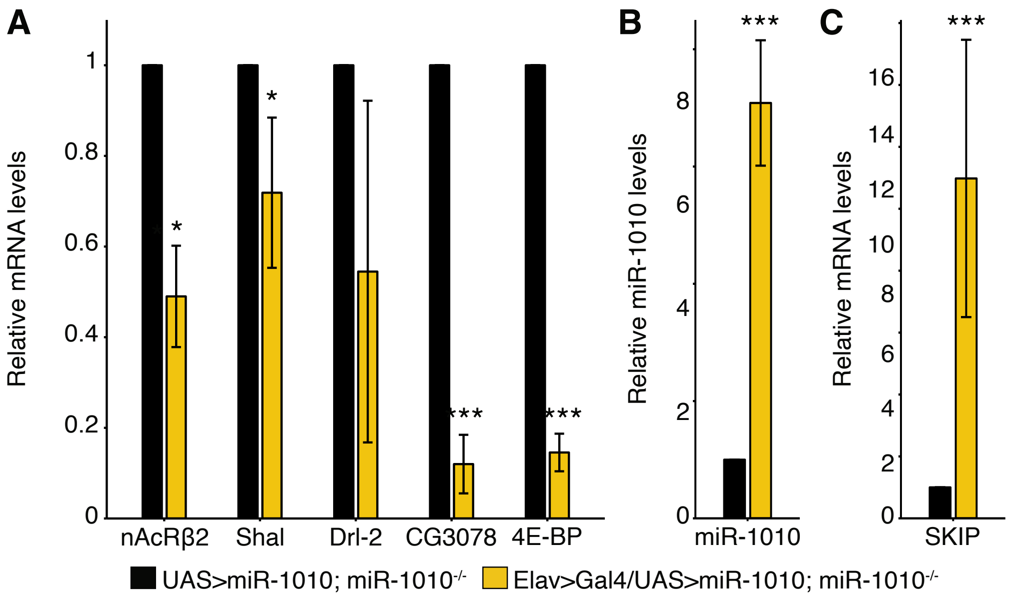

Supplementary Figure S3. MiR-1010 overexpression rescues the lack of miR-1010
(A-C) Relative expression level (RT-qPCR) for nAcRβ2 (A), Shal (A), Drl-2 (A), CG3078 (A), 4E-BP (A), miR-1010 (B) and SKIP (C) in rescue experiments where miR-1010 is restored and expressed under the Elav>Gal4 driver (yellow) in comparison to the non-induced UAS>miR-1010 (black). All values are means ± SD (*P<0.05, **P<0.01, ***P<0.001, n = at least 9 for each experiment).

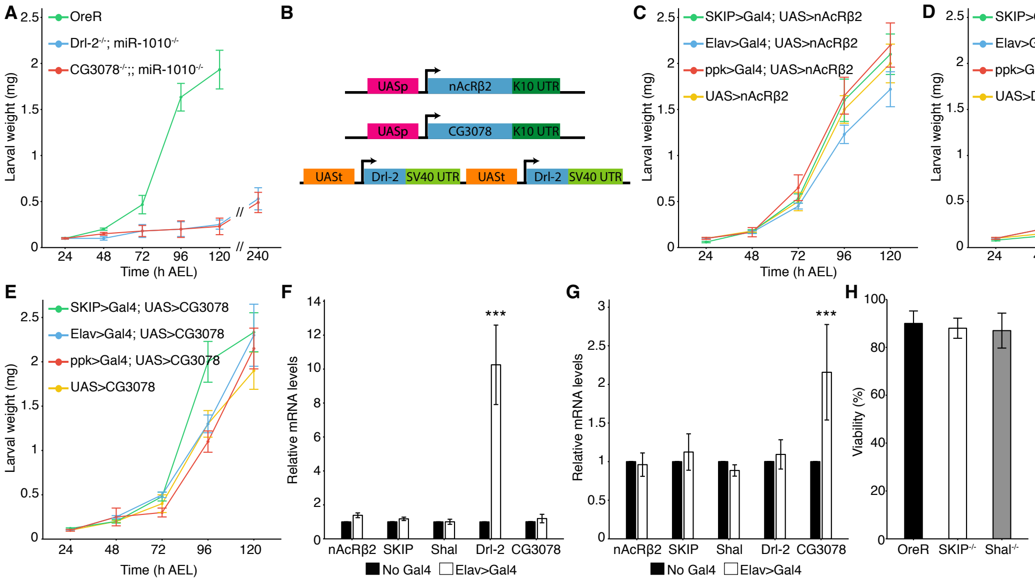

Supplementary Figure S4. Knockdown and overexpression of Drl-2 and CG3078

(A) Growth curves of larvae mutant for Drl-2 (blue) and CG3078 (red) as compared to OreR (green).

(B) Overexpression constructs generated for nAcRβ2, CG3078 and obtained for Drl-2.

(C-E) Growth curves of larvae overexpressing nAcRβ2 (**C**), Drl-2 (**D**) and CG3078 (**E**).

(F) Transcripts levels (RT-qPCR) in Drl-2 overexpressed by Elav>Gal4 (white) relative to non-induce UAS>Drl-2 (black) at 24h AEL.

(G) Transcripts levels (RT-qPCR) in Drl-2 overexpressed by Elav>Gal4 (white) relative to non-induce UAS>CG3078 (black) at 24h AEL.

(H) Adult viability tested in OreR (black), SKIP$^{-/-}$ (white) and Shal$^{-/-}$ (grey). All values are means ± SD (*P<0.05, **P<0.01, ***P<0.001, n = at least 9 for each experiments).

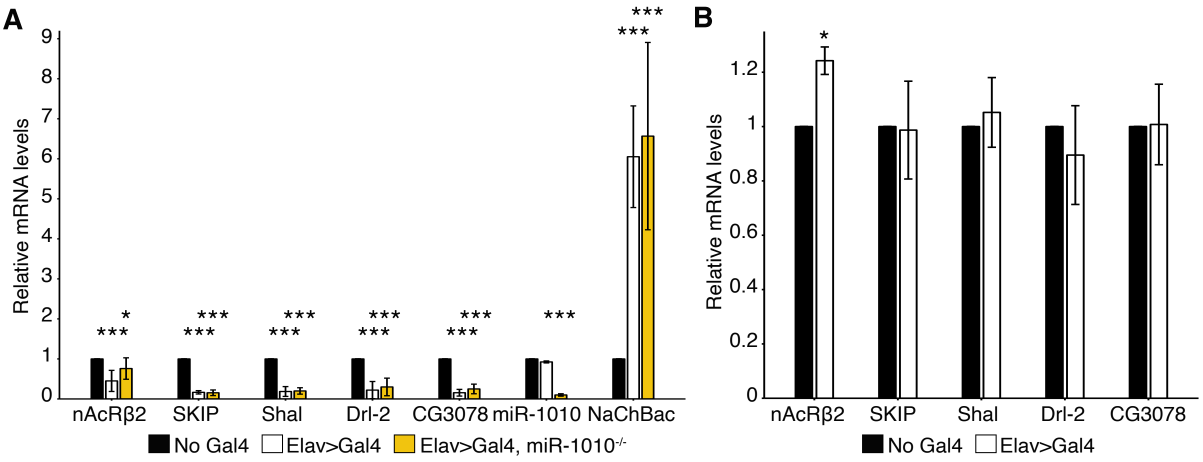

Supplementary Figure S5. Effect of promoting or inhibiting activity on SKIP/miR-1010
(A) Transcripts levels (RT-qPCR) in NaChBac overexpressed by Elav>Gal4 (white) and Elav>Gal4, miR-1010$^{-/-}$ (yellow) relative to non-induce UAS>NaChBac (black) at 24h AEL.
(B) Transcripts levels (RT-qPCR) in Kir2.1 overexpressed by Elav>Gal4 (white) relative to non-induce UAS>Kir2.1 (black) at 24h AEL. All values are means ± SD (*P<0.05, **P<0.01, ***P<0.001, n = at least 9 for each experiments).

Predicted consequential pairing

```
Position 1947-1953 in nAcRβ2 3’UTR  5’ ...AAACAUUUCGUUGACAGGUGAAU...
                                                         |||||||
                          miR-1010  3’  GACGUUUACCUUGCUAUCCACUUU
```

Supplementary Figure S6. Predicted target sequence in the nAcRβ2 3’UTR. Predicted target sequence of miR-1010 in the nAcRβ2 3’UTR was obtained from the TargetScanFly v6.2.

```
Alignment of Sequence_1:  [nAcRb2_3UTR.xdna] with  Sequence_2:
[1stBASE_sequencing_nAcRb2.xdna]

Similarity : 390/409 (95.35 %)

Seq_1   1     CCCTGGCCAATCATATTTTTCGTACCAGTAATTGGCTTTATTAATTAAACAAATTATTAT   60
              ||||||||||||||||||||||||||||||||||||||||||||||||||||||||||||
Seq_2   1     CCCTGGCCAATCATATTTTTCGTACCAGTAATTGGCTTTATTAATTAAACAAATTATTAT   60

Seq_1   61    GATTGTGTTTTCATTTGGTTGTTGTTATGTGTTCAATGTCGTTGTTGGTTGATTGTTGGT   120
              ||||||||||||||||||||||||||||||||||||||||||||||||||||||||||||
Seq_2   61    GATTGTGTTTTCATTTGGTTGTTGTTATGTGTTCAATGTCGTTGTTGGTTGATTGTTGGT   120

Seq_1   121   TTGATTGTTTGACTGTTTGACCGTTTGATGGCTCGTGTATCTGTCGGATCGAGTGGGTAG   180
              |||| |||||||||||||||||| | ||||||||||||||||||| ||||||||| ||||
Seq_2   121   TTGACTGTTTGACTGTTTGACCGCTCGATGGCTCGTGTATCTGTCTGATCGAGTGTGTAG   180

Seq_1   181   CGATAGCCGAGAGATATCGACAGGTGGAAATTGTGTAAACATTTCGTTGACAGGTGAAGT   240
              ||||||||||||||||||||||||*||||||||||||||| ||||||||||||||*||||
Seq_2   181   CGATAGCCGAGAGATATCGACAGGGGGAAATTGTGTAAACGTTTCGTTGACAGGTTAAGT   240

Seq_1   241   TATGTATTTCGATACTCGTGTCGTTGACCGTGTTTTTCCTGCTCTTGGCCGAAATTATCC   300
              |||||| ||||||||||| |||| |||||| | || |||||||||||||||||||||||
Seq_2   241   TATGTACTTCGATACTCGAGTCGGTGACCGCGGTTCTCCTGCTCTTGGCCGAAATTATCC   300

Seq_1   301   CCCCAACCTCGTTGGCCGTGCCCCTGCTGGGCAAGTACCTTCTCTTTACCATGATACTCG   360
              ||||||||||| |||||| |||||||||||||||| ||||||||||||||||||||||||
Seq_2   301   CCCCAACCTCGGTGGCCGCGCCCCTGCTGGGCAAGCACCTTCTCTTTACCATGATACTCG   360

Seq_1   361   TCTCGCTATCCGTTTGGACGACGGTCTGCGTGCTGAACATCCATTTCAG   409
              |||||||||||| ||||||||| ||||||||||||||||||||||||||
Seq_2   361   TCTCGCTATCCGATTGGACGACTGTCTGCGTGCTGAACATCCATTTCAG   409
```

→	Sequencing primer
↔	miR-1010 binding site
↔	5′ homology arm
↔	3′ homology arm
TGG	PAM site
*	CRISPR modifcations

Supplementary Figure S7. Genomic sequencing of the nAcRβ2 3'UTR in the nAcRβ2$^{\Delta1010}$.

The nAcRβ2 3'UTR was sequenced in nAcRβ2$^{\Delta1010}$ homozygous larvae (sequence 2) and aligned against the unmodified nAcRβ2 sequence (sequence 1). The PAM site (orange) has been modified to prevent further modification. The miR-1010 binding site (red) has been has been modified as shown in sequence 2 (nucleotide in green). The modifications introduced by CRISPR are shown with an *. 5' and 3' homology arms used in the donor ssODN are depicted in purple and blue, respectively.

A

Genes	Gene full names
Dfd	Deformed
Ftz	Fushi tarazu
Prd	Paired
Mad	Mothers against dpp
Adf-1	Adh transcription factor-1
DsxF	Doublesex (female isoform)
DsxM	Doublesex (male isoform)
dl	Dorsal
Tll	Tailless

B

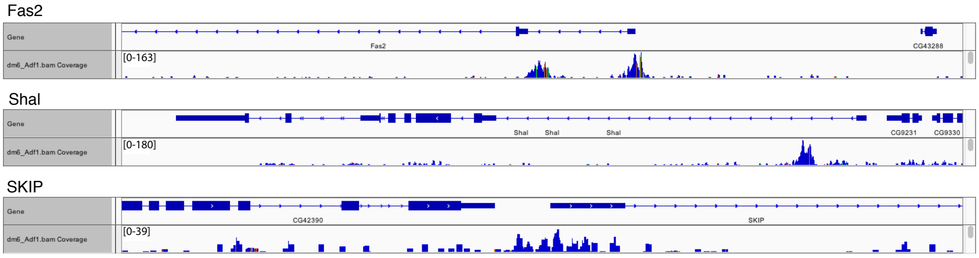

Supplementary Figure S8. Adf-1 binding sites in SKIP and Shal regulatory regions
(A) Putative transcription factor binding sites identified in SKIP and Shal regulatory regions by PROMO (http://alggen.lsi.upc.es/cgi-bin/promo_v3/promo/promoinit.cgi?dirDB=TF_8.3).
B ChIP-seq for Adf-1 results visualized with Integrative Genomics Viewer in Fas2 (top), Shal (middle) and SKIP (bottom) gene regions.

Supplementary Movie S1. SKIP/miR-1010 expression across embryogenesis
Confocal imaging of embryos expressing a membrane marker (UAS>Gap43::mVenus) driven by SKIP>Gal4. Top panel shows a ventral view while the bottom panel shows a lateral view. SKIP/miR-1010 are expressed in the CNS, in axons emanating from the CNS and at neuromuscular junctions.

Supplementary Note: Modeling the miR-1010 - SKIP pathway

Here, we outline the model used to simulate the behaviour of the miR-1010/SKIP network. As outlined in Fig. 4, the network can be decomposed into a negative feedback loop (involving miR-1010) acting on nAcRβ2 and a negative feedforward loop (involving SKIP) acting on the membrane potential. We analyse each loop independently and then combine into a final model. Importantly, we are modelling the long-term behaviour of the network (on the order of hours), not the short-term response of individual membrane potentials (sub-second time scales). In effect, we are modelling the ensemble average of the system response to a stimulus, rather than the response of individual elements. This is because we are focused on the long-term readout of this network and its effects on growth, not on the local changes that are occurring. Further, we ignore spatial effects - *e.g.* diffusion of the chemical components between membrane and nucleus - that can also be potentially important. Essentially, we assume protein transport occurs on much faster time scales than the production and interaction of the network components.

Negative feedback loop: nAcRβ2 - miR-1010 interaction

We consider the interactions between nAcRβ2 (concentration denoted by [R]) and miR-1010 (concentration denoted by [M]) interactions, as outlined in Fig. 4. We model the positive feedback of nAcRβ2 on itself using a Hill function (this saturates after some time, as required for biological relevance). MiR-1010 suppresses production of nAcRβ2. Using standard mass-action kinetics, the concentration of nAcRβ2 and miR-1010 can be approximated by

$$\frac{dR}{dt} = d \cdot \frac{1}{[M]} \cdot \frac{[R]^2}{[R]^2 + R_0^2} - \xi[R] \quad (1)$$

$$\frac{dm}{dt} = c[R] - \zeta[M] \quad (2)$$

for nAcRβ2 and miR-1010 respectively. c and d are the production rates of miR-1010 and nAcRβ2 respectively. ζ and ξ are the degradation rates of miR-1010 and nAcRβ2 respectively. R_0 is the concentration dependence of the positive feedback - small R_0 corresponds to stronger positive feedback. The production of miR-1010 depends on the level of nAcRβ2, where here we ignore the intermediate steps involving Adf-1.

Using the substitutions $t = \tau/\xi$, $[R] = R_1 r$, and $[M] = cR_1/\zeta$, where $R_1 = \sqrt{d\zeta/c\xi}$, we can re-express these equations in terms of the dimensionless components r (representing the effective nAcRβ2 concentration) and m (representing the effective miR-1010 concentration)

$$\frac{dr}{d\tau} = \frac{1}{m} \cdot \frac{r^2}{r^2 + \tilde{r}^2} - r \quad (3)$$

$$\frac{dm}{d\tau} = \frac{r - m}{\tilde{\zeta}} \quad (4)$$

where $\tilde{r} = R_0/R_1$ and $\tilde{\zeta} = \zeta/\xi$.

Now, in steady-state it is straightforward to see that either $r_{ss} = 0$ (if $\tilde{r} > 1$) or $r_{ss} = \sqrt{1 - \tilde{r}^2}$ (if $\tilde{r} < 1$, where for any reasonable parameter values $\tilde{r}$ is positive definite).

Therefore, the long-time behaviour of the system can be described by a single dimensionless parameter, which represents the balance between positive feedback on nAcRβ2 (determined by R_0) and suppression by miR-1010 (incorporated within R_1). Effectively, if

the suppression of nAcRβ2 by miR-1010 is strong then the state returns to a low nAcRβ2 expression state. Note that this network, as modelled here, is independent of SKIP and Shal.

Of course, the form of the above equations is unrealistic as levels of nAcRβ2 are not likely to go to zero in the relevant cells. Further, at very low miR-1010 levels the rate of production of nAcRβ2 can be unrealistically large. Therefore, there is likely a basal level of production. Including such basal production alters the equation for nAcRβ2:

$$\frac{dr}{d\tau} = \frac{1}{m} \cdot \frac{r^2}{r^2 + \tilde{r}^2} - r + \tilde{\kappa} \tag{5}$$

where $\tilde{\kappa}$ represents the basal production levels of nAcRβ2. For small $\tilde{\kappa}$, this results in a small increase in the effective point at which the system switches into a high expression state. However, it does not alter the fundamental behaviour of the network.

Synaptic potential response

nAcRβ2 boosts the synaptic potential V. In contrast, Shal [Sh] - which is activated by nAcRβ2 via Adf-1 - acts to temper the synaptic potential. This tempering effect is magnified by SKIP [Sk] - which is also activated by nAcRβ2 via Adf-1 - as it induces Shal to switch into its slow inactivation mode. For simplicity, we again ignore the intermediate steps and take nAcRβ2 to be regulating expression of both Shal and SKIP .

To model this system we consider the following system:

$$\frac{d[Sh]}{dt} = b[R] - \eta[Sh] \tag{6}$$

$$\frac{d[Sk]}{dt} = c[R] - \mu[Sk] \tag{7}$$

$$\frac{dV}{dt} = v_{hs} + a\frac{[R]^m}{[Sh](1+\alpha[Sk])} - \nu V \tag{8}$$

where v_{hs} is the homeostatic response that ensures the resting potential, $V_{rest} = \gamma/v_{hs} \approx -70meV$. Upon activation of nAcRβ2 , the potential increases $\approx +40meV$. The action of Shal and SKIP act to bring the average potential back to its normal range. Recall that the potential V represents the average across many synapses, and is not modelling the response of individual synapses - which occurs on much faster time scales.

Again, we can use substitutions to non-dimensionalize these equations: $[Sh] = (bR_1/\eta)s$; $[Sk] = (cR_1/\mu)k$; and $V = V_0 v + v_{hs}/\nu$:

$$\frac{ds}{d\tau} = \frac{r-s}{\tilde{\eta}} \tag{9}$$

$$\frac{dk}{d\tau} = \frac{r-k}{\tilde{\mu}} \tag{10}$$

$$\frac{dv}{d\tau} = \tilde{a}\frac{r^m}{s(1+\tilde{\alpha}k)} - \tilde{\nu}v \tag{11}$$

where $\tilde{\nu} = \nu/\xi$, $\tilde{\eta} = \eta/\xi$, $\tilde{\nu} = \nu/\xi$, $\tilde{a} = \eta a R_1^{m-1}/bV_0\xi$ and $\tilde{\alpha} = \alpha c R_1/\mu$.

Taking $m = 2$ (which promotes a strong membrane potential response to increase in nAcRβ2), and considering steady-state we find $k_{ss} = r_{ss}$, $s_{ss} = r_{ss}$ and

$$v_{ss} = \frac{\tilde{a}}{\tilde{\nu}} \cdot \frac{r_{ss}}{1+\tilde{\alpha}r_{ss}} \tag{12}$$

So, if nAcRβ2 returns to low expression levels after stimulation, then the average potential also reduces back towards its normal levels. However, if nAcRβ2 is in a stable active state (e.g. due to reduced miR-1010 suppression) then the average potential remains high, though its levels are tempered by the negative feedforward interaction via Shal and SKIP. Eq. 12 has a Michaelis-Menten like form for potential response as a function of nAcRβ2 concentration. Therefore, this simple model reveals clearly how the competing components of the network ensure stable membrane potential output.

Furthermore, we can see how the action of SKIP helps to ensure the system is robust. As a simplification, we consider how the voltage varies in steady-state depending on nAcRβ2 expression using Eq. 12. If there is variability such that $r = r_{ss} + \delta r$, then the variability in $v = v_{ss} + \delta v$ is given by

$$\frac{\delta v}{v_{ss}} = \frac{1}{1+\tilde{\alpha} r}\left(\frac{\delta r}{r_{ss}}\right) \tag{13}$$

Therefore, variations in the levels of nAcRβ2 are buffered by SKIP, to ensure that the membrane potential remains relatively unperturbed.

Complete network

Combining the above two sections, we have the following five differential equations describing nAcRβ2 (r), miR-1010 (m), Shal (s), SKIP (k) and the average membrane potential response (v):

$$\frac{dr}{d\tau} = \frac{1}{m}\cdot\frac{r^2}{r^2+\tilde{r}^2} - r \tag{14}$$

$$\frac{dm}{d\tau} = \frac{r-m}{\tilde{\zeta}} \tag{15}$$

$$\frac{ds}{d\tau} = \frac{r-s}{\tilde{\eta}} \tag{16}$$

$$\frac{dk}{d\tau} = \frac{r-k}{\tilde{\mu}} \tag{17}$$

$$\frac{dv}{d\tau} = \tilde{\alpha}\frac{r^m}{s(1+\tilde{\alpha}k)} - \tilde{\nu} v \tag{18}$$

We have eight dimensionless parameters. However, five of these ($\tilde{\zeta}$, $\tilde{\mu}$, $\tilde{\eta}$, $\tilde{\nu}$ and $\tilde{a}$) do not alter the general behaviour of the system - they mainly alter the final expression levels of different components. For the voltage potential to be stable at low concentration levels requires $m > 1$, and we take $m = 2$ as a simple approximation.

We are effectively left with two key parameters: $\tilde{r}$ and $\tilde{\alpha}$. $\tilde{r}$ determines the stable state of nAcRβ2. If $\tilde{r} < 1$, then nAcRβ2 can stably exist at high concentration levels. If $\tilde{r} > 1$ then nAcRβ2 exists at low concentration levels. Therefore, $\tilde{r} = R_0\sqrt{c\xi/d\zeta}$ is the key parameter determining the system state. $\tilde{\alpha}$ plays an important role in determining the strength of the SKIP input into suppression of the voltage potential. This plays a crucial role in determining the time scale for the system to return to equilibrium after stimulation.

Through these simple networks we see how the regulation of SKIP and miR-1010 from the same locus can provide powerful regulatory potential. By simultaneously expressing factors that temper the synaptic potential (via SKIP) and suppress the activator of the synaptic potential, nAcRβ2 (via miR-1010), the system has developed a rapid response mechanism to stop over-activation of the membrane potential.

This model is obviously a simplification of the underlying network. It excludes, for example, Adf-1, and diffusion is not incorporated. However, despite its simplicity, the model can explain a number of our observations: (1) why miR-1010$^{-/-}$ is lethal but miR-1010$^{+/-}$ and SKIP$^{-/-}$ are not; and (2) the time delays in growth for miR-1010$^{+/-}$ and SKIP$^{-/-}$. Further, the model predicts that SKIP is serving to buffer variability in nAcRβ2 levels, to ensure robust membrane potential. The model encapsulates how both negative feedforward and feedback loops can work in concert to ensure careful control of membrane potential.

An interesting point here is that miR-1010 likely evolved later than SKIP, yet it is absolutely essential for function whereas SKIP is at least partially redundant. One reason could be the following: (1) initially nAcRβ2 response was tempered by Shal; (2) SKIP evolved to further amplify the tempering action of Shal, but in of itself it was not essential; (3) increased production of nAcRβ2 was favoured as it leads to more rapid response to stimuli; (4) with more nAcRβ2 , the system has the potential to switch into a permanently "on" state, and so a repressor (i.e. miR-1010) became necessary to down-regulate nAcRβ2 response. Subsequently, the levels of nAcRβ2 could reach levels such that the potential would stay high even with dampening by Shal and SKIP unless miR-1010 was present - so the system became dependent on miR-1010 but not SKIP.

Simulation details

For the model output shown in Fig. 4, we use the following parameters:

Parameter	WT value
$\tilde{\zeta}$	1
$\tilde{\mu}$	1.1
$\tilde{\eta}$	0.9
$\tilde{\nu}$	0.2
$\tilde{a}$	1
m	1.8
$\tilde{\alpha}$	2
$\tilde{r}$	2

For miR-1010$^{+/-}$ we take $\tilde{r} = \sqrt{2}$, miR-1010$^{-/-}$ we take $\tilde{r} = 0$, and for SKIP$^{-/-}$ we take $\tilde{\alpha} = 0$ with all other parameters kept constant. Note, in the case of the mutants we effectively change the interaction strengths of the appropriate elements, rather than remove the elements from the model. This is for convenience and has the same functional effect on the voltage output. Eqs. 14-18 were solved in Matlab using ode45 for $\tau = 0$ to $\tau = 100$. To prevent singularities in the equations, we adapt Eq. 14 and Eq. 18 such that $\frac{dr}{d\tau} = \frac{1}{m+\delta} \cdot \frac{r^2}{r^2+\tilde{r}^2} - r$ and $\frac{dv}{d\tau} = \tilde{a}\frac{r^m}{(s+\epsilon)(1+\tilde{\alpha}k)} - \tilde{\nu}v$ respectively where $\delta = 10^{-3}$ and $\epsilon = 0.5$. All components were started at 0, with the exception of $r(t = 0) = 1$, corresponding to a stimulus resulting in a burst of nAcRβ2. In the Supplementary Theory Figure below we outline the behaviour of each component of the network for the four conditions considered. Further, we also show the behaviour expected in a weaker - but not null - miR-1010 mutant with $r = 0.9$. This system is just within the regime where nAcRβ2 concentration levels remain "on". This provides an additional prediction of the model - it is likely that miR-1010$^{+/-}$ are only just viable. Further perturbation of miR-1010 concentrations could switch the behaviour of the system into the nAcRβ2 "on" state, resulting in lethality.

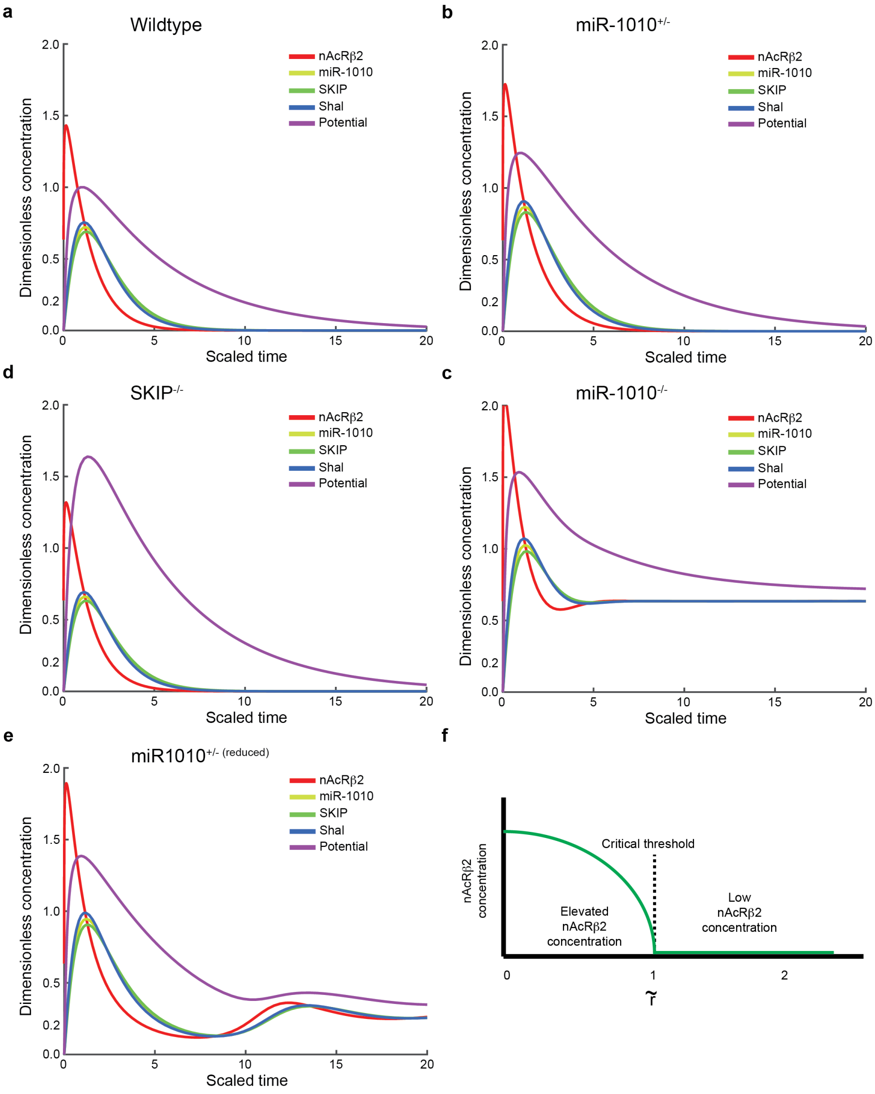

Supplementary Theory Figure. (**a-e**) Concentration of the five elements of the model (r, m, k, s and v) as a function of time with initial conditions and parameters as described in text (note v is actually a dimensionless potential but for ease of nomenclature we refer to it as a concentration here). For (**e**) we take $\tilde{r} = 0.9$, which is just below the critical threshold for the receptor to enter the "on" state (see **f**). Note that the mutants are modelled by turning off the interactions with targets, rather than removing the appropriate protein from the model. Within this modelling framework these two approaches have the same effect. (**f**) Cartoon depiction of the dependence of nAcRβ2 on the parameter $\tilde{r}$. The critical point is at $\tilde{r} = 1$.
